# No evidence of widespread decline of snow cover on the Tibetan Plateau over 2000–2015

**DOI:** 10.1038/s41598-017-15208-9

**Published:** 2017-11-07

**Authors:** Xiaoyue Wang, Chaoyang Wu, Huanjiong Wang, Alemu Gonsamo, Zhengjia Liu

**Affiliations:** 1 State Key Laboratory of Remote Sensing Science, Institute of Remote Sensing and Digital Earth, Chinese Academy of Sciences, Beijing, 100101 China; 2 University of the Chinese Academy of Sciences, Beijing, 100049 China; 3 Institute of Geographical Sciences and Natural Resources Research, Chinese Academy of Sciences, Beijing, 100101 China; 4 Department of Geography and Planning, University of Toronto, 100 St. George St, Toronto, ON M5S 3G3 Canada

## Abstract

Understanding the changes in snow cover is essential for biological and hydrological processes in the Tibetan Plateau (TP) and its surrounding areas. However, the changes in snow cover phenology over the TP have not been well documented. Using Moderate Resolution Imaging Spectroradiometer (MODIS) daily snow products and the Interactive Multi-sensor Snow and Ice Mapping System (IMS) data, we reported daily cloud-free snow cover product over the Tibetan Plateau (TP) for 2000–2015. Snow cover start (SCS), melt (SCM) and duration (SCD) dates were calculated for each hydrological year, and their spatial and temporal variations were analyzed with elevation variations. Our results show no widespread decline in snow cover over the past fifteen years and the trends of snow cover phenology over the TP has high spatial heterogeneity. Later SCS, earlier SCM, and thus decreased SCD mainly occurred in the areas with elevation below 3500 m a.s.l., while regions in central and southwestern edges of the TP showed advanced SCS, delayed SCM and consequently longer SCD. The roles of temperature and precipitation on snow cover penology varied in different elevation zones, and the impact of both temperature and precipitation strengthened as elevation increases.

## Introduction

Snow cover is an important component of land cover and the rapid accumulation and melting of snow makes it one of the most active natural materials on the Earth’s surface^1^. Snow cover parameters, including snow-covered days, snow cover areas, and snow depth, are the most important input parameters for climate, hydrological and ecological models^2^. Snow cover is mostly controlled by atmospheric conditions and its anomalies may in turn potentially influence the large-scale atmospheric conditions by changing heat and moisture fluxes^3^. In addition, snow cover has high albedo^4^ and the variation in snow cover is a key indicator of climate variability and change^5,6^. Although the response of snow cover to climate change is complicated, as snow formation and melt are closely related to a temperature threshold of 0 °C^7^, it is one of the most predictable climate change indicators^8^. Therefore, monitoring spatial and temporal patterns of snow cover in various regions has received great attention in the scientific community^9–13^.

According to the recent report from Intergovernmental Panel on Climate Change (IPCC), global mean surface temperature showed a warming trend of 0.85 °C over the period 1880 to 2012. In the Northern Hemisphere (NH), 1983–2012 was likely the warmest 30year period of the last 1400 years^14^. In response to the warming trend, the spatial extent and the duration of snow cover (SCD) have both decreased significantly in the NH^10^. However, there exists significant difference in the response of snow cover to global warming in different regions. For example, the snowmelt date advanced remarkably in Europe and Central Asia during the past three decades, whereas it remained stable over North America^12,15^. Similarly, contrast changes in snow cover were observed in northern middle latitudes, where SCD was longer by 9.74 days, and high latitudes with SCD being shorter by 5.57 days from 2001–2014^16^. Based on the daily snow observation data from meteorological stations, Ke *et al*. examined the variability of snow cover phenology in China during 1952–2010^17^. They found that snow cover significantly started later and melted earlier with the increasing temperature, but SCD did not show significant trend within this time span. In addition, the snowfall of western China exhibited an upward trend with large spatiotemporal variations during the past half century^18^.

The Tibetan Plateau (TP) is the highest region in terms of altitude in the world and has been characterized as the amplifier for global climate change^19^. TP has abundant snow resources in China, and snow cover is closely related to biological and hydrological processes in the TP and its surrounding areas^20^. Furthermore, the plateau’s snow cover plays an important role in regulating the Asian monsoon^21^. Thus, information of temporal and spatial patterns of snow cover over the TP is of great significance for scientific endeavors and management applications. In the context of global warming, the temperature also rose obviously on the TP^22^, resulting in substantial changes in snow cover. Based on daily snow depth data acquired from meteorological stations, Zhu *et al*. found that the snow depth over the TP decreased after 2002^23^. Shen *et al*. also found that the extent of snow cover on the plateau was reduced by 5.7% from remote sensing data over the period 1997–2012^24^. Nevertheless, the study from Huang *et al*. showed that both the duration and depth of snow increased on the southwest edge and in the southeast part of the TP through combining optical remote sensing snow cover products and passive microwave snow depth data^25^. Thus, further work is still needed to investigate the change in snow cover on the TP. In addition, previous studies mainly focused on changes in the extent, duration and depth of snow cover^26,27^, while spatial and temporal patterns of snow cover start (SCS) and melt (SCM) dates of the TP have not been well understood. Studies have systematically analyzed the variability of snow cover phenology in China^17^ and the Northern Hemisphere^12^, using meteorological stations. However, these stations distribute unevenly and predominantly located in low-altitude areas of the TP. SCS and SCM also have widespread ecological implications^16^. Earlier snow melt can lead to major alterations in the timing and volume of spring snowmelt runoff and consequently affects the soil water storage^28^. Moreover, changes in snow cover phenology may also influence the terrestrial ecosystems through altering thaw and freeze dates of the soil^29^. And they may also influence spring time land surface phenological trend over the TP^30^ as the climate conditions and topographic features of the TP are complex and variable.

In this study, Moderate Resolution Imaging Spectroradiometer (MODIS) daily snow products and the Interactive Multi-sensor Snow and Ice Mapping System (IMS) data were used to produce daily cloud-free snow cover product over the TP for 2000–2015. Then, the spatial and temporal patterns of SCD, SCS and SCM of the TP in different elevation zones were analyzed, and the drivers of SCS and SCM were identified using climate data.

## Results

### No widespread decline in snow cover over the time duration

**Figure 1:**
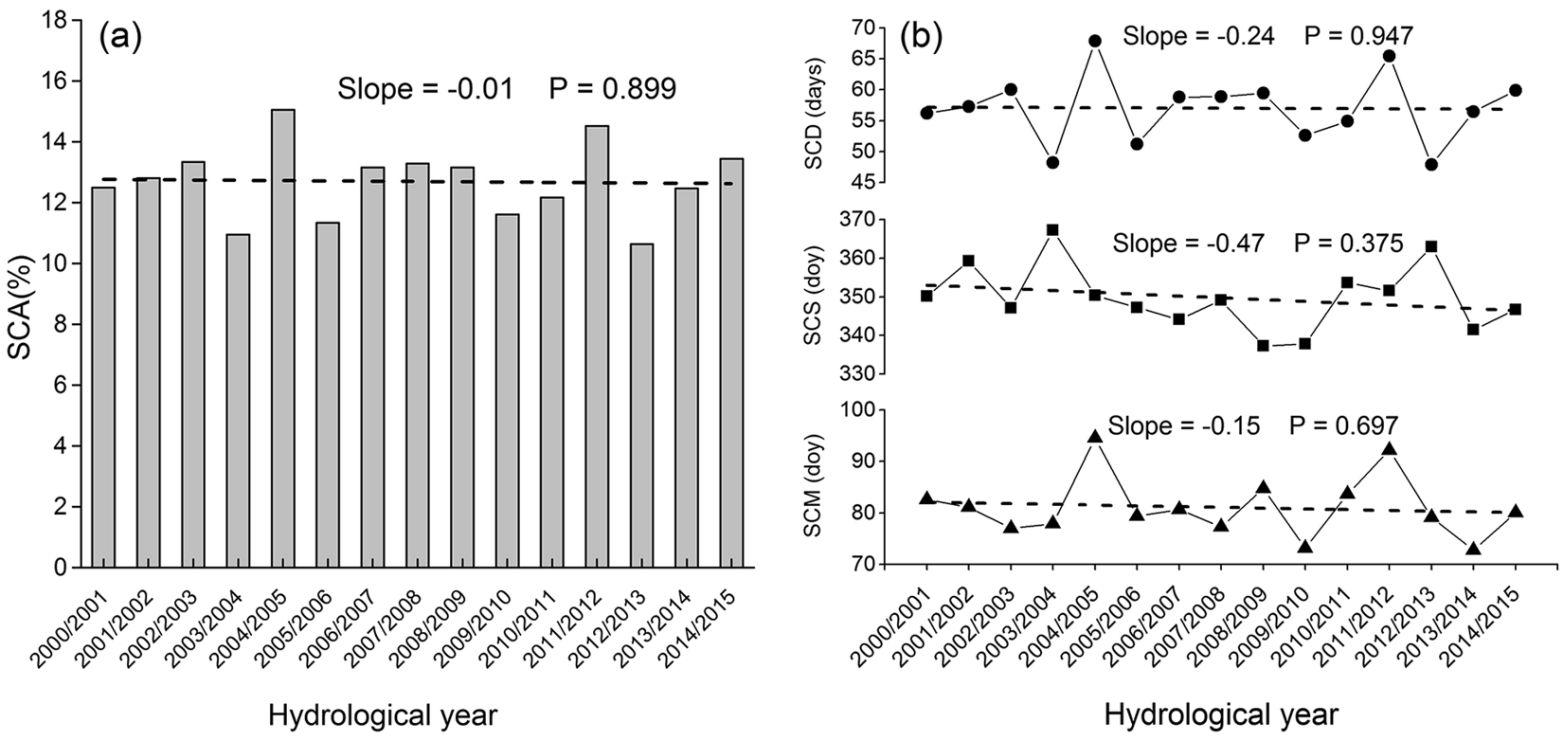
Interannual variabilities of (**a**) snow-covered area (SCA) and (**b**) duration (SCD), start (SCS), and melt (SCM) of snow cover over the whole Tibetan Plateau (TP) from 2000 to 2015.

Based on the meteorological data, the TP showed an accelerated warming trend after 1998^22^. However, as shown in Fig. 1a, the snow-covered area (SCA) over the plateau was relatively stable during the hydrological year 2000/2001 to 2014/2015, suggesting that snow cover of the TP did not show widespread decline over the past 15 years. The changes in snow cover phenology over the whole plateau also provided evidence that there is no significant decrease in snow cover (Fig. 1b). The SCD showed a decreasing trend with a rate of −0.24 day per year for the time 2000–2015. However, this tendency was not significant (P = 0.947). Similarly, although both the snow cover start and melt became earlier from 2000 to 2015 (Slope = −0.47 d/y and −0.15 d/y, respectively), the changes were not significant neither (P > 0.1).

### Spatial changes in snow cover phenology

**Figure 2:**
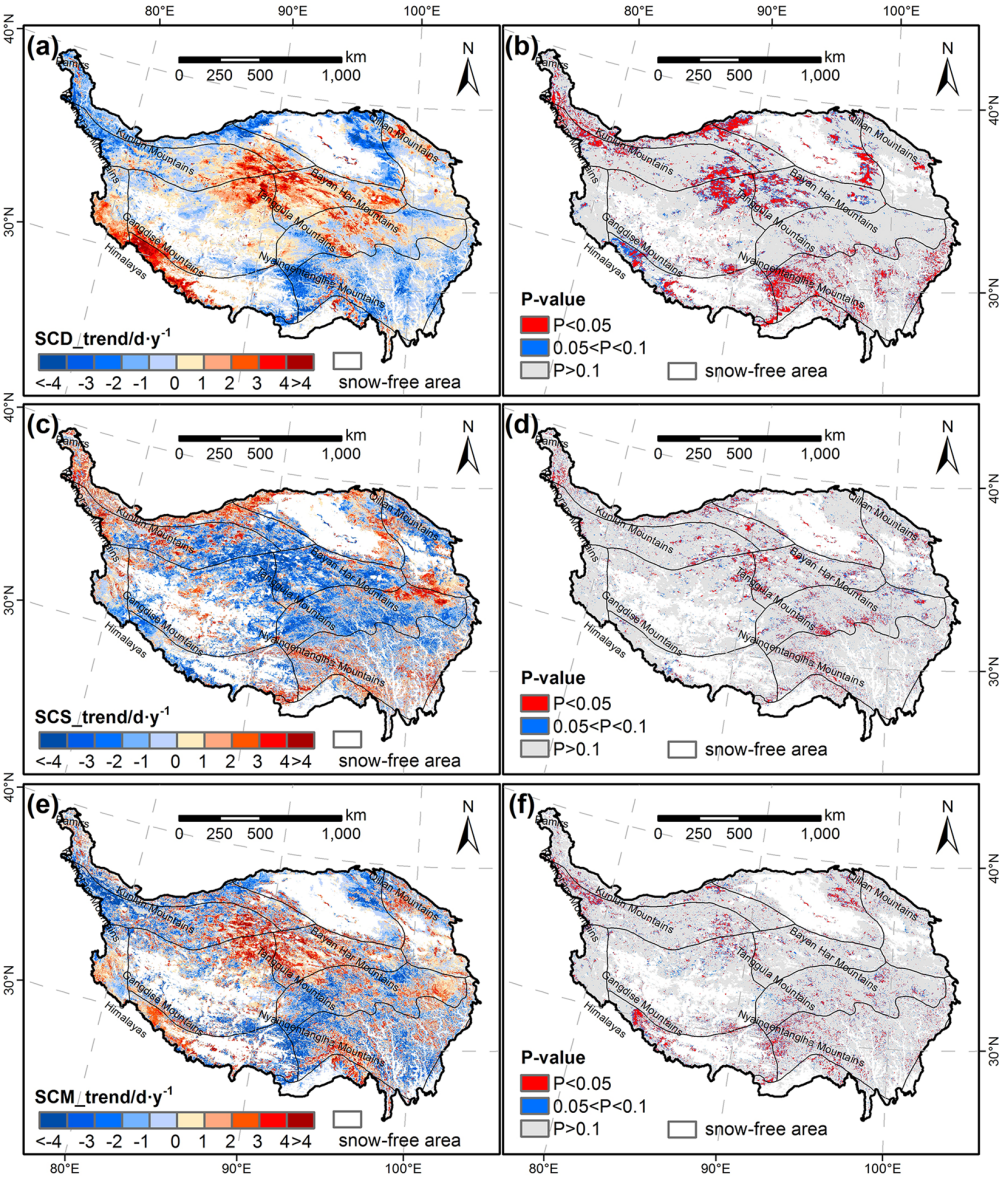
Trend of duration (SCD), start (SCS), and melt (SCM) of snow cover for 2000–2015. (**a**) and (**b**) Represent the trend of SCD and its p-values; (**c**) and (**d**) represent the trend of SCS and its p-values; (**e**) and (**f**) represent the trend of SCM and its p-values. The maps were generated by ArcGIS 10.2, URL: http://support.esri.com/Products/Desktop/arcgis-desktop/arcmap/10-2-2#overview.

To explore the spatial pattern of changes in snow cover phenology, trend analysis was performed at each pixel. High spatial heterogeneity was observed in trends of SCD, SCS and SCM (Fig. 2). The SCD decreased (slope < 0) in 52.7% of the whole plateau during the fifteen hydrological years, primarily in western Sichuan-eastern Tibet zone and the northern part of the TP. The largest decrease in SCD was found about 4 d/y in the western part of Nyainqentanglha Mountains and northwestern and southeastern borders of Qaidam Basin. The decreases were significant (P < 0.1) in 11.0% of the total area. However, we also observed a large region (47.3%) with increasing SCD (slope > 0), and the increase was significant in 7.9% of the area. Particularly, the increasing rates of the southwestern and central parts of the plateau were above 4 days per year. The regions with advancing SCS accounted for about 58.6% of the TP, and 6.5% of the total area, which were scattered in the central TP, showed a significant advance. Moreover, SCS was delayed in 41.4% of the plateau, but only 3.8% of the area was significant, mainly located in the western part of Himalayas. There were 6.6% regions of the TP with a significant trend of early SCM, and only 4.7% area with a significant trend of late SCM, while 88.6% of the plateau showed no significant trend. These early trends primarily distributed in the northwestern mountain areas and western part of Nyainqentanglha mountains, and the late trends were scattered in the central plateau.

**Figure 3:**
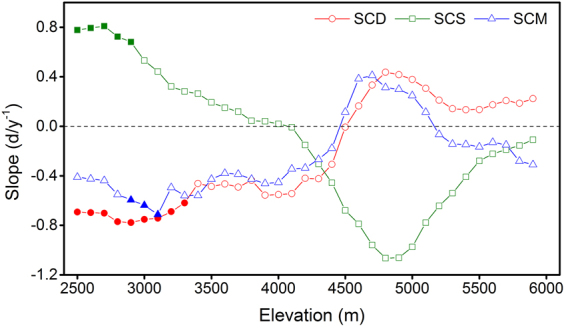
Trend of duration (SCD), start (SCS), and melt (SCM) of snow cover in different elevations for 2000–2015. The solid symbols indicate that changes are significant at 90% confidence levels.

As the topography of the Tibetan Plateau is complicated, we calculated slope of SCD, SCS, and SCM during 2000–2015 based on different elevations with 100 m interval to further illustrate the spatial heterogeneity of the trends of snow cover phenology (Fig. 3). The SCD decreased below the elevation of 4600 m a.s.l and the slope generally declined as the elevation increased. In particular, these decreases were significant below 3400 m a.s.l. However, the SCD increased above 4600 m a.s.l and exhibited no evident trend with increasing elevation. SCS showed delayed trends below 4000 m a.s.l and the slope decreased with the elevation. We found that SCS advanced above 4000 m a.s.l, and these advancing trends gradually strengthened as the elevation increased between 4000 and 4900 m a.s.l, and then became weaker with further increase of elevation. SCM with earlier trends were found below 4500 m a.s.l and above 5200 m a.s.l, while later trend was shown in the elevation range between 4500 and 5200 m a.s.l.

### Climate controls on SCS and SCM

To investigate the responses of snow cover phenology to climate change, we analyzed the relationship between SCS and autumn temperature and precipitation at the pixel level using the partial correlation. And the same method was conducted to analyze the relationship between SCM and spring temperature and precipitation.

**Figure 4:**
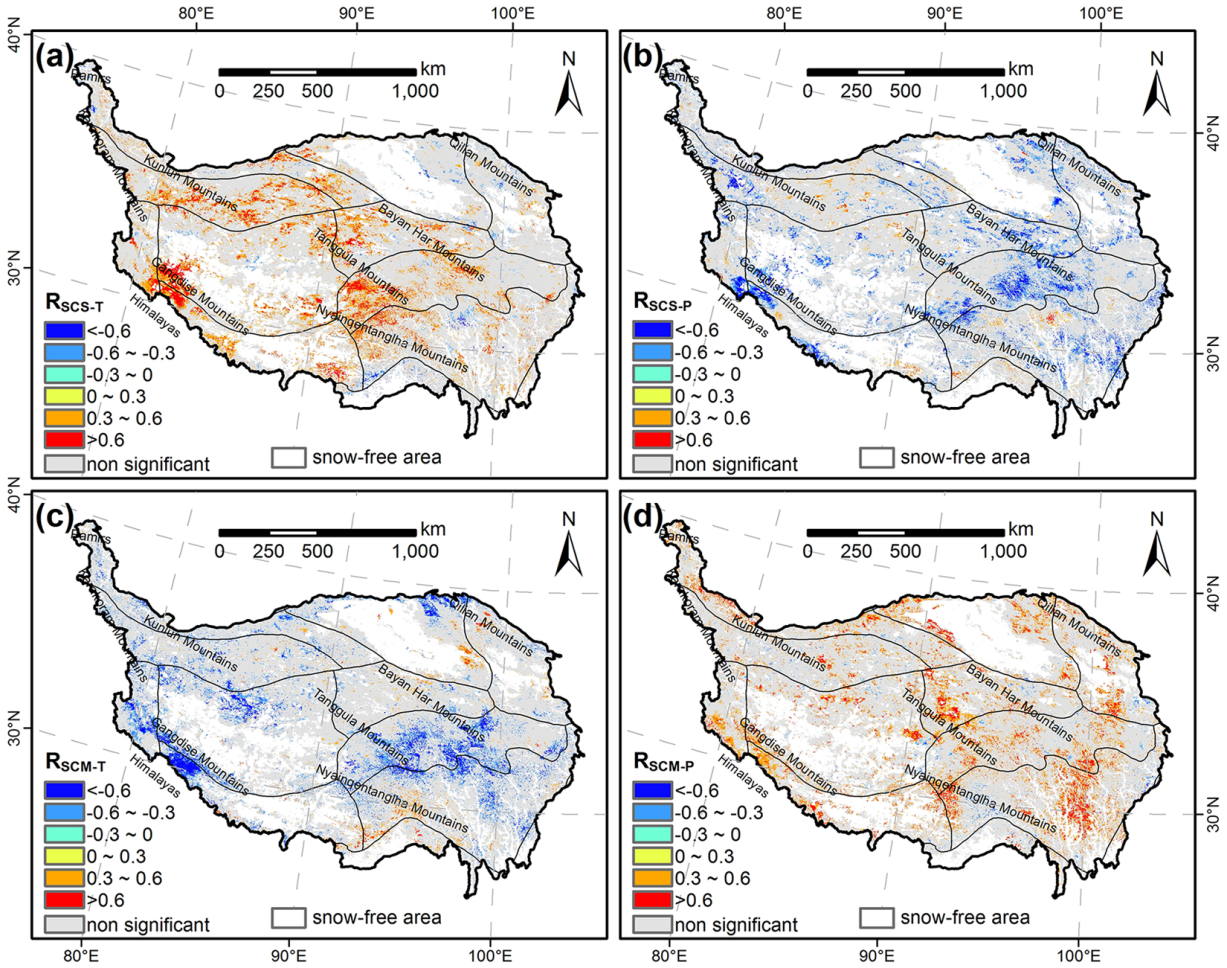
Climate controls on snow cover start (SCS) and melt (SCM) date. Partial correlation between (**a**) SCS and autumn temperature (mean value for September–November), (**b**) SCS and autumn precipitation (sum for September–November), (**c**) SCM and spring temperature (mean value for March-May), and (**d**) SCM and spring precipitation (sum for March-May). The maps were generated by ArcGIS 10.2, http://support.esri.com/Products/Desktop/arcgis-desktop/arcmap/10-2-2#overview.

As shown in Fig. 4a, a significant positive correlation between the SCS and autumn temperature was found in 16.6% of the study area, mainly located in the north and central plateau and southwestern margins. We also observed an inverse effect of autumn temperature on SCS, i.e., a small area (1.6%) in the south of Nyainqentanglha Mountains showed negative correlation between SCS and autumn temperature. The correlation also revealed that the autumn precipitation may also control SCS in several regions (Fig. 4b). We found a significant negative correlation between the SCS and autumn precipitation in the Southern Qinghai high-cold zone, Golog-Nagqu high-cold zone and Western Sichuan montane zone, and western borders of the TP, accounting for 12.5% of the area. However, a small proportion (1.8%) of areas in the southern border and northern part of the plateau showed a positive correlation.

SCM was also controlled by spring temperature (Fig. 4c) and precipitation (Fig. 4d). The SCM was significantly correlated with temperature in about 15.8% of the plateau. 13.5% of the area showed negative correlation, primarily in Golog-Nagqu high-cold zone and northern and southwestern borders of the plateau. While positive relationship between SCM and temperature was observed in a small region (2.3%) in southern part of Nyainqentanglha Mountains and eastern border of the Qaidam Basin. The regions with significantly positive correlation between SCM and precipitation mainly scattered in mountain areas, accounting for 15.7% of the TP. However, the significantly negative was only 1.2% of the area.

**Figure 5:**
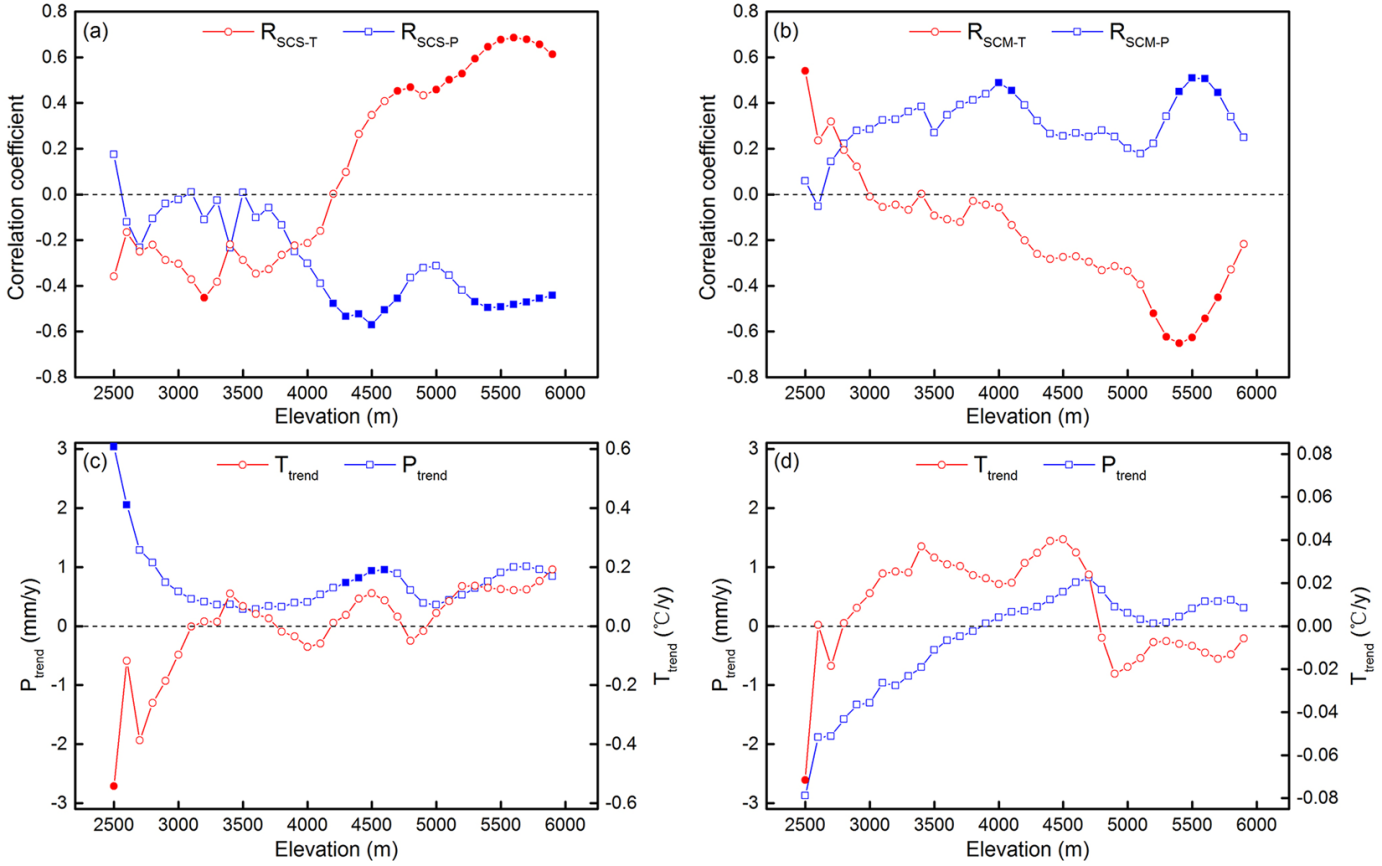
Partial correlation coefficient between (**a**) snow cover start (SCS) and autumn temperature and precipitation, and partial correlation coefficient between (**b**) snow cover melt (SCM) and spring temperature and precipitation in different elevations. Trend of (**c**) autumn temperature and precipitation, and (**d**) spring temperature and precipitation for 2000–2015 in different elevations. The solid symbols indicate that the correlations or trends are significant at 90% confidence levels.

To further examine the spatial pattern of impacts of climate factors on SCS and SCM, we also analyzed their relationships with the elevation variations. The correlation coefficients (R) between SCS and autumn temperature and precipitation were shown in Fig. 5a. The relationship between SCS and temperature was negative at the elevation below 4300 m a.s.l, and these values did not show a clear trend. When the elevation exceeded 4300 m a.s.l, the relationship became positive, and the impact of temperature became stronger. The SCS negatively correlated with precipitation except for the elevation below 2500 m a.s.l. The significantly negative correlations were observed between 4200 m a.s.l and 4700 m a.s.l, and above 5300 m a.s.l. Figure 5b showed that the negative effects of temperature on SCM occurred mainly above 3000 m a.s.l. This impact strengthened as altitude increased, but weakened above 5000 m a.s.l. The SCM positively correlated with precipitation almost in all elevation zones. Moreover, the significant relationship occurred in the elevation ranges of 4000–4200 m a.s.l and 5300–5600 m a.s.l.

We also detected the trends of temperature and precipitation in different elevation zones (Fig. 5c and d). The autumn temperature showed declined trend below 3000 m a.s.l, and fluctuated near the zero line between 3000 and 5000 m a.s.l, and then increased as further increase in elevation. The autumn precipitation exhibited a continuous increase trend, and these increases were significant below 2600 m a.s.l and between 4300 and 4700 m a.s.l. The spring temperature increased at an elevation range of 3000 to 4800 m a.s.l. The precipitation in spring decreased below 3900 m a.s.l and showed increasing trends above this altitude.

## Discussion

In this study, we systematically analyzed the spatial and temporal patterns of snow cover phenology and its response to climate factors at pixel level and for different elevation zones based on the MODIS snow products with cloud removal. Snow cover phenology showed high spatial heterogeneity over the Tibetan-Plateau (Supplementary Fig. S1). Snow cover is high in the mountainous regions, where snow cover starts earlier and melts later and its duration is relatively longer. However, later starting, earlier melting and relatively shorter duration of snow cover all indicate rare snowfall in the vast interior of the plateau. This is in line with previous studies reported elsewhere^20,26,31,32^. The distribution of snow cover over the TP is mainly controlled by atmospheric and topographic conditions. Snowfall is the solid form of precipitation and a necessary condition for the formation of snow cover. The complex topography of the plateau is also an influencing factor of the spatial distribution of snow cover. At higher elevation mountainous, lower air temperature and more precipitation create favorable conditions for the formation and maintenance of snow cover^26^. While, due to strong shielding from these huge mountains, snow cover is relatively scarcer in the most regions of the interior of the TP^20^.

The trends in snow phenology may be more important to understand snow dynamics in response to climate change. Many previous studies confirmed that the spatial extent and the duration of snow cover over the high latitude regions of Northern Hemisphere (NH) have both decreased remarkably due to global warming^10,33–35^. However, the SCA did not show a significant decrease during the past fifteen years in the TP (Fig. 1a). This finding is consistent with the previous study made by Huang *et al*.^36^. Furthermore, we conducted further analysis from the perspective of snow cover phenology. In the context of global warming, late SCS, early SCM and thus shortened SCD would occur^12^. Nevertheless, such trends were not significant over the whole Tibetan Plateau (Fig. 1b). One possible reason could be that even with global warming, there is no reason for snow cover to decrease when snowfall is increasing and temperature remains negative during the snow season^31,37^. Gonsamo *et al*. also observed no trends in snow cover change in the entire northern hemisphere for 1980–2012^8^. This is expected as snow formation is related with temperature threshold of 0 °C which has not changed substantially during the recent global warming. In addition, the highly spatial heterogeneity of these trends across the TP may be the other reason why the snow cover phenology did not show significant trend over the whole plateau. The regions with obviously decreased SCD, late SCS and early SCM were mainly located in the lower areas. It is possible that areas with lower altitude has relatively higher temperature, thus the snow cover phenology would be more sensitive to the increased temperature. In addition, the SCM above 5200 m a.s.l showed earlier trends, but such trends were not significant.

Snow cover phenology is sensitive to changes in meteorological variables, especially to temperature and precipitation. Temperature mainly had a positive impact on SCS but a negative impact on SCM, accounting for 91.2% and 85.4% of all significantly controlled areas, respectively. The decreased temperature could increase the probability of snowfall and is favorable for the maintenance of snow cover, which would cause early SCS in autumn. However, higher temperature would trigger snow melt earlier in spring. The negative correlation between SCS and autumn precipitation was observed in most significantly controlled areas (87.4%), while the SCM showed a positive relationship with spring precipitation in 92.9% of the significantly controlled areas. In the beginning of snow season, the temperature is usually below the frozen point, thus the precipitation probably occurred as snowfall. Therefore, the increased precipitation causes earlier SCS, and vice versa. Similarly, more snowfall would delay SCM in spring.

Previous studies like Bi *et al*.^38^ and Morán-Tejeda *et al*.^39^ found that altitude is the major factor in determining the roles of temperature and precipitation on snow cover variability. Our results also showed that the effects of temperature and precipitation on snow cover phenology varied in different elevation zones. In the study of Morán-Tejeda *et al*.^39^, temperature is the major factor controlling snow cover below a threshold altitude, and precipitation becomes the controlling factor above this threshold. However, in our results, both temperature and precipitation significantly affected SCS and SCM in the relatively higher elevations, but did not show significantly correlation in the lower regions. The reason may be that our study area, Tibetan Plateau, is much larger than Switzerland, and the topography of the TP is much more complicated. In addition, our results were also different from the study of Bi *et al*.^38^. The reason may be that the climate condition is varied over the whole plateau, but the Upper Heihe River Basin is a small part of the TP.

## Methods

### Study area

**Figure 6:**
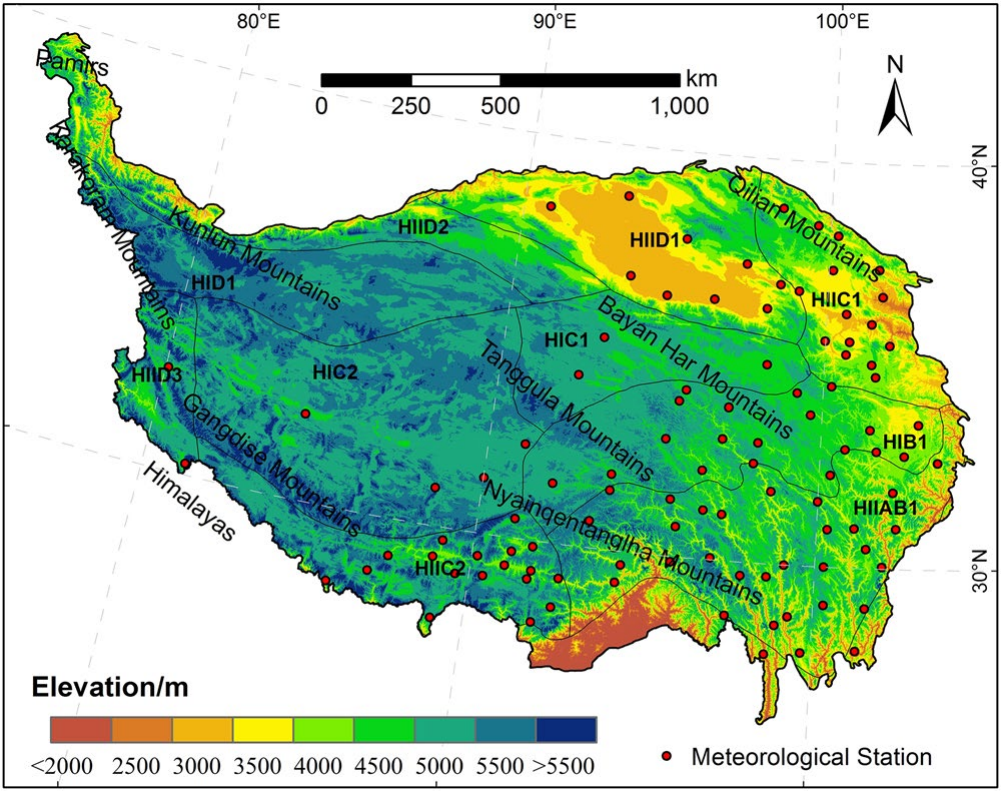
Location and physiographical regions of the Tibetan Plateau. HIB1, Golog-Nagqu high-cold shrub-meadow zone. HIC1, Southern Qinghai high-cold meadow steppe zone. HIC2, Qangtang high-cold steppe zone. HID1, Kunlun high-cold desert zone. HIIA/B1, Western Sichuan-eastern Tibet montane coniferous forest zone. HIIC1, Eastern Qinghai-Qilian montane steppe zone. HIIC2, Southern Tibet montane shrub-steppe zone. HIID1, Qaidam montane desert zone. HIID2, Northern slopes of Kunlun montane desert zone. HIID3, Ngari montane desert zone. The maps were generated by ArcGIS 10.2, http://support.esri.com/Products/Desktop/arcgis-desktop/arcmap/10–2–2#overview.

The Tibetan Plateau (26°00′12′′N-39°46′50′′N and 73°18′52′′E-104°46′59′′E) is located in the southwest of China, with an average elevation of over 4000 m and an area of approximately 2.57 × 10^6^ km^2^ (Fig. 6). It is the highest and largest plateau in the world, which is also called “Third Pole” or the “roof of the world”. Snow cover over the TP is a vital water source in western China. Many large rivers, such as the Yalu Tsangpo, Yangtze, Yellow Rivers, to mention but few have their headwaters there^31^. The unique geographical conditions of TP create unique ecological environment characteristics of the plateau. Therefore, to better investigate the spatial pattern of snow cover phenology, we divided the TP into ten eco-geographical zones (Table S1) based on the thermal conditions, moisture regimes and topographic features^40^.

### Snow cover data

The Moderate Resolution Imaging Spectroradiometer (MODIS) daily snow products MOD10A1 and MYD10A1 (MODIS Terra/Aqua Snow cover Daily L3 Global 500 m SIN GRID V005) were obtained from the National Snow and Ice Data Center (NSIDC) for the time span of 1 September 2000 to 31 August 2015. However, since Aqua satellite began to deliver data in July 2002, we only used MOD10A1 for dates before July 2002. The products are in the sinusoidal projection with 500 m spatial resolution. Previous studies have confirmed that the overall accuracy of MODIS snow products reaches more than 90% in clear-sky condition^41^. However, the extent of cloud contaminated pixels in the data can significantly limit their applications.

Thus, the Interactive Multi-sensor Snow and Ice Mapping System (IMS) was used to remove cloud pixels in MODIS snow cover products. This dataset covers the entire Northern Hemisphere and started to deliver data since 4 February 1997^42^. Since the inception of IMS, the snow cover charts have been produced daily at a nominal resolution of 24 km. Beginning in 23 February 2004, IMS introduced a higher resolution daily product with a nominal resolution of 4 km. In this study, IMS data of 24 km cell size from 1 September 2000 to 22 February 2004 and that of 4 km cell size from 23 February 2004 to 31 August 2015 were used. All the data can be downloaded freely from the NSIDC (http://nsidc.org/).

### ***In-situ*** temperature, precipitation and snow depth

In order to evaluate the accuracy of climate data and snow cover products, monthly mean temperature and total precipitation and daily snow depth of 101 meteorological data over the Tibetan Plateau in 2010 were obtained from China Meteorological Data Service Center (http://data.cma.cn/).

### Grid climate data

Temperature and precipitation data used in this study were calculated from the China Meteorological Forcing Dataset, which were developed by the Data Assimilation and Modeling Center for Tibetan Multi-spheres, Institute of Tibetan Plateau Research, Chinese Academy of Sciences^43^. This dataset provides near surface temperature and precipitation rate with starting from 1979 with high spatial (0.1° × 0.1°) and temporal (3 h) resolutions. Monthly mean temperature and total precipitation were calculated for 2000‒2015. Both grid temperature (R^2^ = 0.93, RMSE = 2.6 °C) and precipitation (R^2^ = 0.98, RMSE = 5.2 mm) data were highly correlated with observations from meteorological stations (Fig. S4).

### DEM data

The Digital Elevation Model (DEM) data was obtained from SRTM 90 m Digital Elevation Database from CGIAR Consortium for Spatial Information (CGIARCSI, http://www.cgiar-csi.org). The nearest-neighbor interpolation was performed to resample the DEM to a spatial resolution of 500 m.

### Procedure for cloud removal

**Figure 7:**
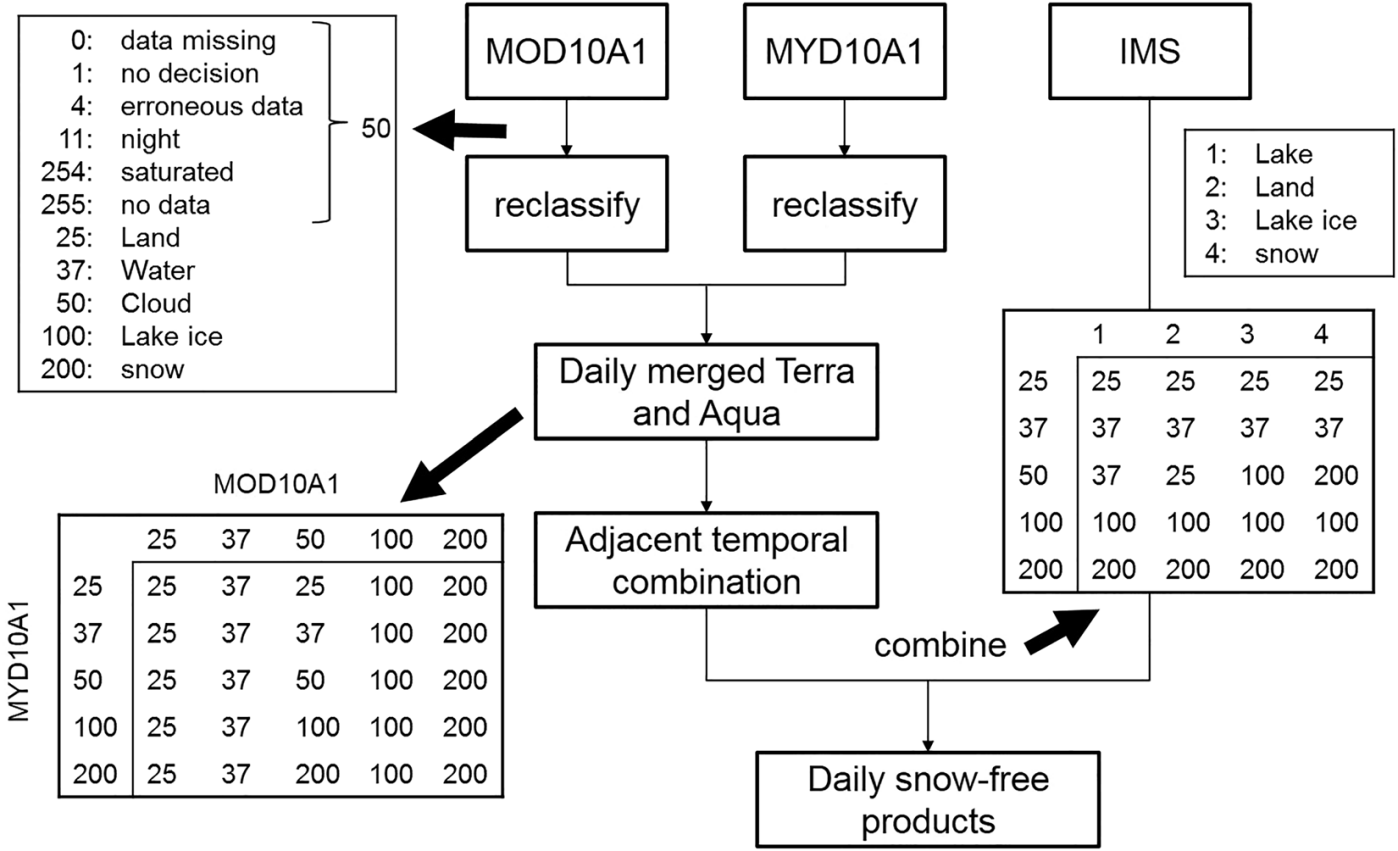
Schematic flow chart of cloud removal.

Based on the daily snow depth measured by meteorological stations, we firstly assessed the original MODIS snow products MOD10A1 and MYD10A1 (Table S2). The overall accuracy of MOD10A1 and MYD10A1 are only 59.4% and 51.1% respectively in all sky conditions due to the impact of clouds, although the accuracy of both data are above 95% in clear sky conditions. Therefore, cloud removal is much important for the application of the MODIS data. In recent years, a lot of methods have been developed to remove clouds^27,44–48^, and we developed an improved algorithm, which included three steps, based on these approaches (Fig. 7). First, MOD10A1 and MYD10A1 were combined to reduce the proportion of cloud pixels. This method has been widely used but could only be applied after 4 July 2002. Since MOD10A1 was not available before 4 July 2002, data before this date was directly transferred to the next step. Second, the data of three successive days was combined to further reduce cloud pixels. If a cloud pixel occurs, products of the prior day and the subsequent day were chosen to replace the cloud pixel in case cloud-free conditions can be found in one of the two observations. Finally, IMS data was used to remove all remaining cloud pixels, i.e. these cloud pixels were substituted by non-cloud pixels (snow, land, water or lake ice) from IMS data. After the procedure of cloud removal, the overall accuracy was significantly improved to 96.6% when compared with *in-situ* measurements (Table S2). Detailed discussion of the algorithm and its effectiveness of cloud removal were provided in Supplementary Information.

### Calculation of snow cover phenology

The snow cover phenology parameters, SCD, SCS and SCM, were calculated based on the daily cloud-free snow product in each hydrological year (1 September to 31 August of the next year) from 2000 to 2015. SCD is calculated by the following equation:

1 $$SCD=\sum _{i=1}^{n}{s}_{i}$$

Where SCD stands for snow cover duration, *n* is the total number of days within a hydrological year, *s*_*i*_ is occurrence of snow cover, with values 1 for snow and 0 for no snow, respectively.

SCS is defined as the first five consecutive days when a pixel is classified as snow and SCM is defined as the last five consecutive days when snow occurs during each hydrological year. This is the easiest way to calculate SCS and SCM, and would avoid the impact of ephemeral snow. Such definition of start and end of snow cover have also been used by Peng *et al*.^12^ and Chen *et al*.^16^.

### Statistical analyses

To assess the snow cover phenology trends and climate factors, the least-squares liner fitting method was adopted, and the slope of the fitting line was calculated for each pixels by equation (2).

2 $$slope=\frac{n\times \sum _{j=1}^{n}\,j\times {k}_{j}-\sum _{j=1}^{n}\,j\sum _{j=1}^{n}{k}_{j}}{n\times \sum _{j=1}^{n}\,{j}^{2}-{(\sum _{j=1}^{n}j)}^{2}}$$

Where *j* is the serial number from 1 to *n*, *n* is the total number of years, *k*_*j*_ is the parameters (SCD, SCS, SCM, temperature and precipitation) of year *j*. And slope < 0 indicates a decreasing tendency, slope > 0 indicates an increasing trend.

When we analyzed the climate controls on snow cover phenology, we calculated partial correlation coefficient between SCS and autumn (September to November) mean temperature and total precipitation, as well as SCM and spring (March to May) mean temperature and total precipitation. In addition, a t-test was performed to reflect the significance level (p-values) of the trend and interannual relationship.

### Data availability

The source of all datasets used in this study has been presented in the Methods section.

## Electronic supplementary material

Supplementary Information
