# Supplementary material for: No evidence of widespread decline of snow cover on the Tibetan Plateau over 2000–2015
Source: Sci Rep. 2017 Nov 7;7:14645. doi: 10.1038/s41598-017-15208-9 (PMC5676715; doi:10.1038/s41598-017-15208-9)
Supplement: Supplementary file 1 — Supplementary Information [file 41598_2017_15208_MOESM1_ESM.pdf]

No evidence of widespread decline of snow cover on the Tibetan Plateau over 2000–2015

Xiaoyue Wang[1,2], Chaoyang Wu[2,3*], Huanjiong Wang[3*], Alemu Gonsamo[4], Zhengjia Liu[3]

1. State Key Laboratory of Remote Sensing Science, Institute of Remote Sensing and Digital Earth, Chinese Academy of Sciences, Beijing 100101, China;
2. University of the Chinese Academy of Sciences, Beijing 100049, China;
3. Institute of Geographical Sciences and Natural Resources Research, Chinese Academy of Sciences, Beijing 100101, China;
4. Department of Geography and Planning, University of Toronto, 100 St. George St., Toronto, ON M5S 3G3, Canada
*Corresponding author: wucy@igsnrr.ac.cn (C.Wu)

Text S1: Spatial patterns of snow phenology.

Spatial patterns of SCD, SCS and SCM were described by their mean values and the standard deviation (sd) over the study period (Fig. S1). Large areas in the TP were covered by snow but distributed very unevenly because of the climate and topographic factors. Regions that had long SCD are mainly located at the northwest edge of the TP while SCD was shorter in the vast interior areas except for places with high mountains. Overall, there were two highest snow cover concentrated areas in the TP. One was Himalayas and Nyainqentanglha Mountains region in the southern edge of the TP. The other was in the western edge of the TP, including Pamirs, KaraKoram Mountains and western Kunlun Mountains. SCD in these regions generally were more than 210 days. In addition, SCD was also substantial long (~120 to 210 days) in western Gangdise, Tanggula, Bayan Har and Qilian Mountains. In the central and eastern plateau, SCD was relatively short with about 60 days. The regions with shortest SCD (less than 10 days) were found in the south of Nyainqentanglha Mountains, the river valleys of southern Tibet, Qangtang area, Qaidam Basin and south regions of Qilian Mountains. We thus defined these regions as snow-free areas, which were not investigated further in the following sections. Overall, most regions had low sd of SCD within 20 days over the study period, accounting for 60.7% of the plateau area. Regions with sd of SCD between 20 and 40 days comprised about 34.1%, and the percentage of sd of SCD higher than 40 days was only 5.3%. In particular, we found that regions with relatively higher sd of SCD mainly located in the southwestern border of the plateau, central of the Golog-Nagqu zone and west of the Qilian Mountains.

The spatial distribution of SCS and its standard deviation had strong heterogeneity (Fig. S1c and d). Snow occurred earlier than day of year (DOY) 260 in regions with high altitudes. With the decrease of altitude, SCS occurred in later dates. In general, SCS was before DOY 320 in most

mountainous areas. However, Ngari zone, the south region of the Kunlun Mountains and the eastern part of TP had late SCS, where snow usually occurred after DOY 365. We found that sd of SCS increased from southeast to northwest. The highest sd of SCS were found at the eastern section of the Kunlun Mountains and the western section of the Gangdise Mountains.

The spatial distribution of SCM showed a contrasting shape to SCS (Fig. S1e and f). Regions with high elevation had the latest SCM dates, and while SCM overall occurred earlier with decreasing elevation. Specifically, snow melting dates were later than DOY 165 in the western borders of the TP, e.g., the west Kunlun Mountains, Nyainqentanglha Mountains, and several high elevation areas in the central plateau. In the vast interior of the TP, SCM ranged between DOY of 60 to 90. The earliest SCM (before DOY 45) were found in the north of Qangtang and south of Qinghai. Most regions had small sd of SCM within 45 days and the regions with largest sd (above 45 days) were located in the south border of the TP.

We further investigated snow cover phenology along the altitude gradient. As shown in Fig. S2, SCS substantially advanced, SCM delayed, and SCD decreased with an increasing altitude. In particular, these trends became much more obviously above the elevation of 5000 m a.s.l.

Text S2: Discussion of the algorithm and its effectiveness of cloud removal.

MODIS daily snow cover products have been widely used to monitor snow cover dynamics for various regions in recent years[1-4]. Unfortunately, cloud contamination greatly limits the application of these products. We firstly applied two previously reported steps: combining Terra and Aqua products and adjacent temporal combination successively[1,5]. However, not all cloud pixels can be removed after these two steps. Some researches chose to increase the days of temporal combination[6,7], but in this way we would reduce the temporal resolution and might thus influence the accuracy of snow phenology date retrieval. Passive microwave sensors can penetrate cloud cover while they also provide the information of snow depth and snow water equivalent. Thus, several studies used passive microwave data to remove cloud pixels[8,9]. However, the spatial resolution of these data is 25 km, which is too coarse compared to MODIS data. This may also lead to rather low spatial resolution in the composite images if a large number of cloud pixels exist. Due to the aforementioned challenges, we proposed a new algorithm which employs the IMS data to remove the residual cloud pixels. This process effectively removed all the cloud pixels and kept higher spatial and temporal resolution as well. Yu et al[10] developed daily cloud-free snow products through combining MODIS Terra-Aqua and IMS, and the overall accuracy of their products is 94%. Our algorithm added adjacent temporal combination, which could further reduce the proportion of cloud pixels, before fusing MODIS and IMS. Consequently our products should have higher accuracy in snow identification.

We took the data of 1 February 2011 as an illustration example for the effectiveness of cloud removal approach. Both MOD10A1 and MYD10A1 had a large number of cloud pixels, accounting for 32% and 43.3% of the whole plateau respectively, and there was no cloud pixel in IMS data (Fig. S3a, b and c). After the combination of MOD10A1 and MYD10A1, the percentages of cloud pixels were reduced to 22.6% (Fig. S3d). The adjacent temporal combination could further eliminate partial cloud coverage. However, there were still 16.3% of the pixels covered by cloud (Fig. S3e). Then, based on the IMS data, all the remaining cloud pixels were removed, and the resulting cloud-free data is shown in figure S3 f. This method effectively curtailed the problem of cloud interference as compared to the original MODIS data. Thus, it is more accurate to use the cloud-free data to

analyze the spatial and temporal patterns of the snow cover phenology over the TP.

Text S3. Accuracy assessment of original MODIS snow products and our daily cloud-free snow map.

We evaluated the accuracy of the original MODIS snow products and our daily cloud-free snow map based on daily snow depth data from meteorological stations. We calculated the snow accuracy and overall accuracy in both clear-sky and all-sky conditions (Table S2). The overall accuracy of both MOD10A1 and MYD10A1 is more than 97% in clear-sky condition. However, it decreases to about 50% in all-sky conditions, because of the effect of cloud. After the process of cloud removal, the overall accuracy was significantly improved to 96.6% in all-sky condition.

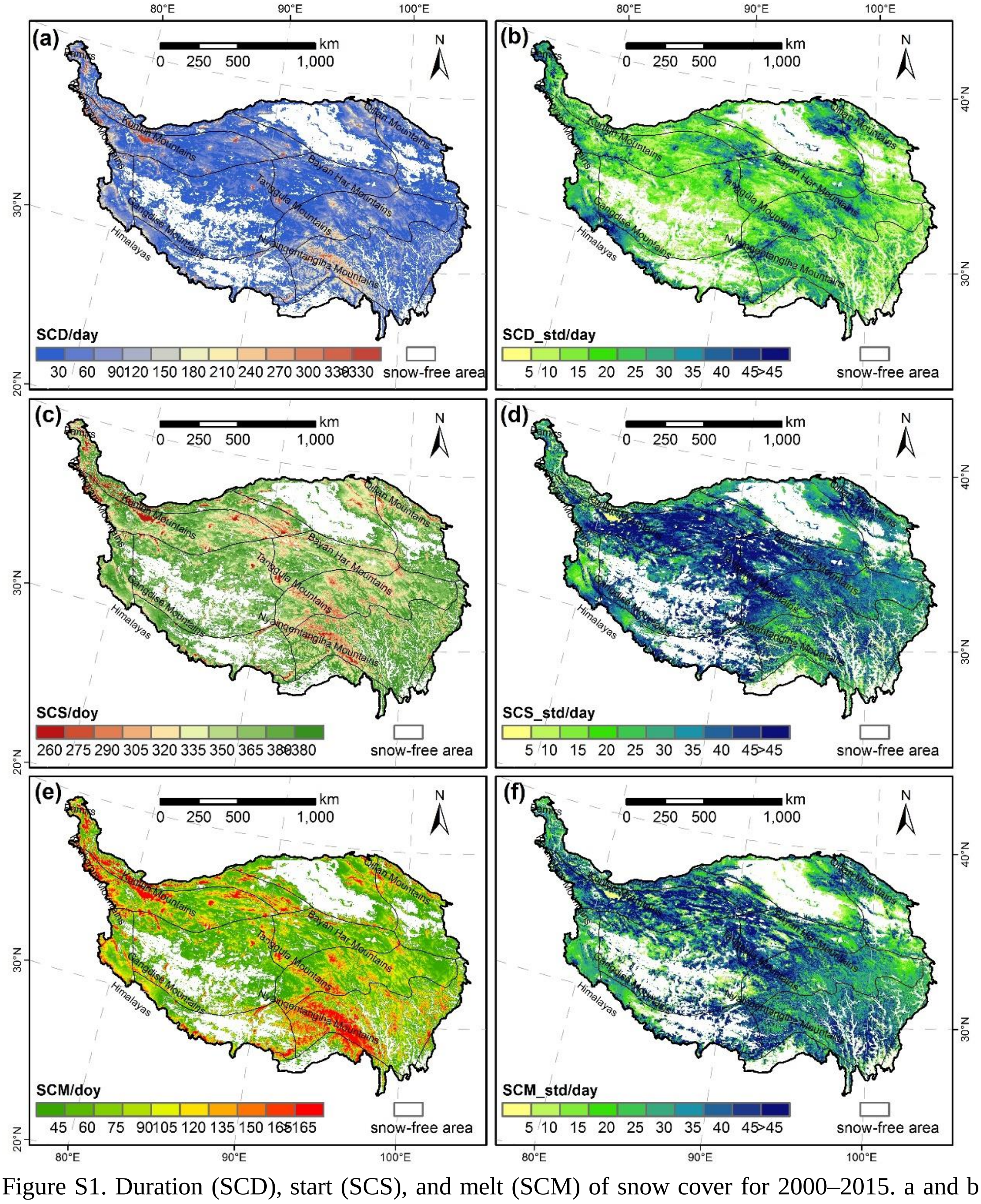

Figure S1. Duration (SCD), start (SCS), and melt (SCM) of snow cover for 2000–2015. a and b

represent SCD and its standard deviation; c and d represent SCS and its standard deviation; e and f represent SCM and its standard deviation. The maps were generated by ArcGIS 10.2, URL: http://support.esri.com/Products/Desktop/arcgis-desktop/arcmap/10-2-2#overview.

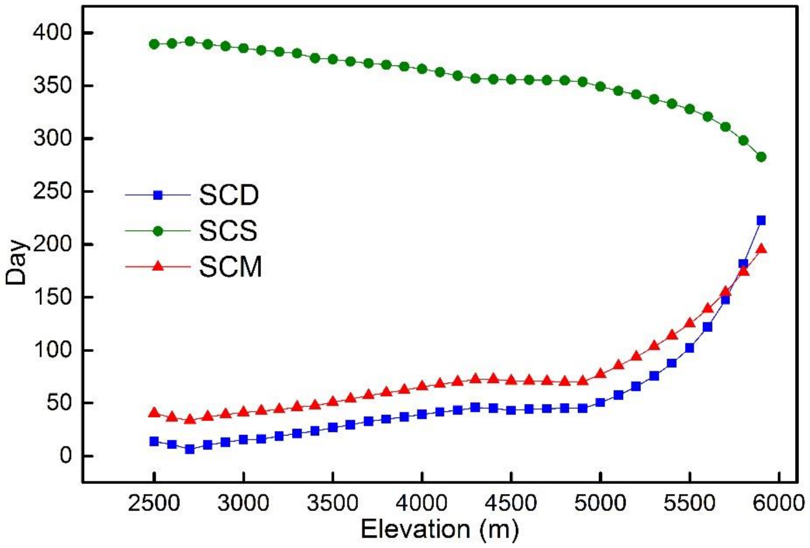

Figure S2. Average duration (SCD), start (SCS), and melt (SCM) of snow cover for 2000–2015 in different elevations.

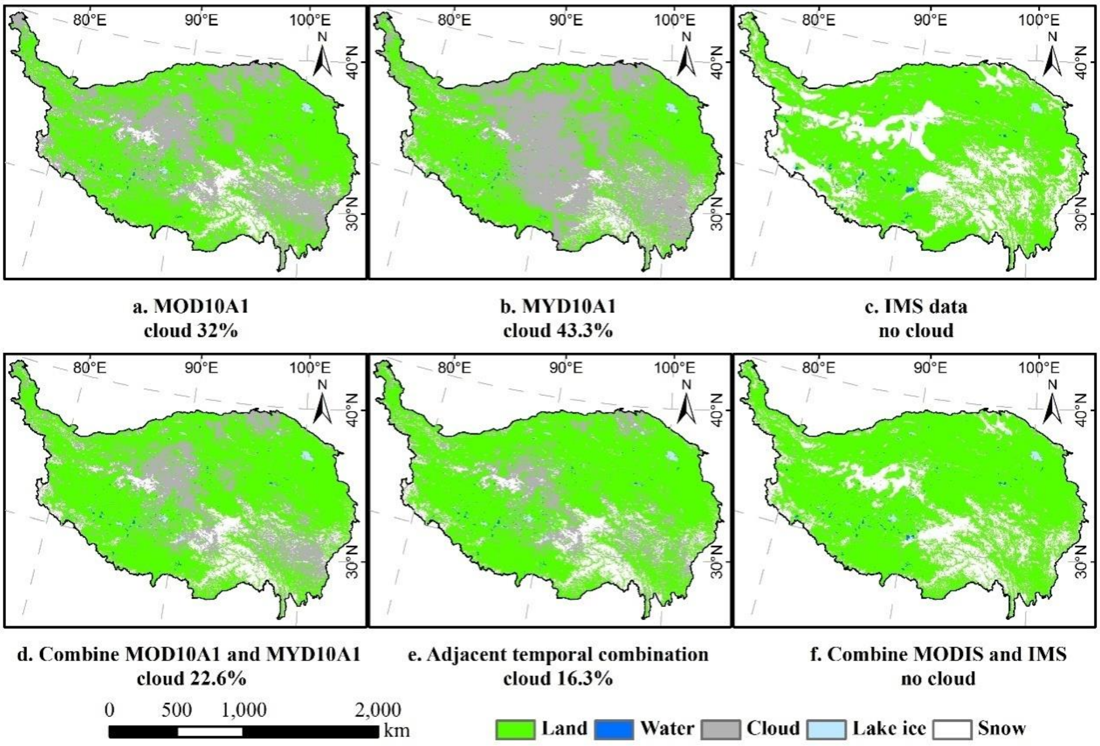

Figure S3. Percentage of cloud pixels at each step in the process of cloud removal. The maps were generated by ArcGIS 10.2, URL: http://support.esri.com/Products/Desktop/arcgis-desktop/arcmap/10-2-2#overview.

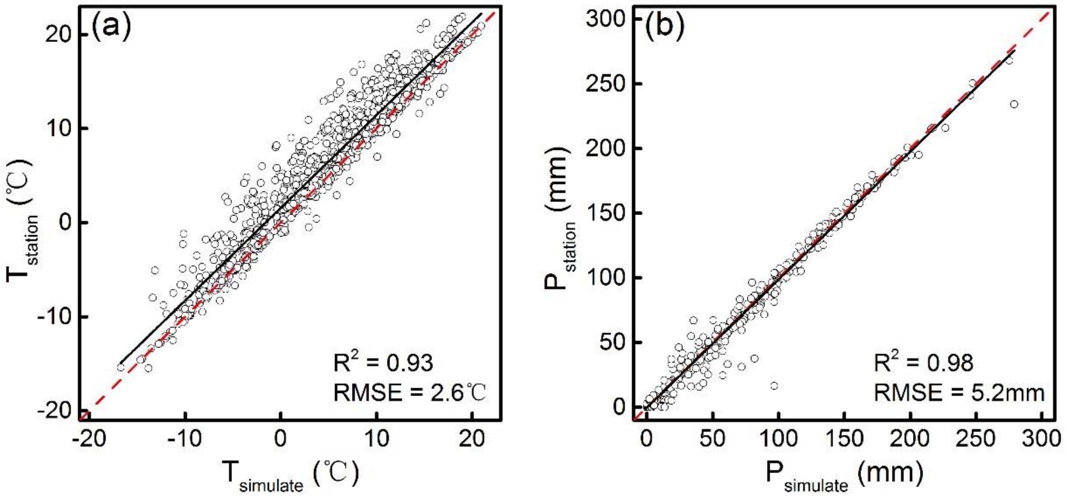

Figure S4. Relationships (a) between grid temperature data and in-situ temperature, (b) between grid precipitation data and in-situ precipitation.

Table S1. Eco-geographical zones of the Tibetan Plateau.

Temperature zone		Humidity region		Eco-geographical zone	
HI	Plateau sub-cold zone	B	Sub-humid zone	HIB1	Golog-Nagqu high-cold shrub-meadow zone
		C	Semi-arid zone	HIC1	Southern Qinghai high-cold meadow steppe zone
				HIC2	Qangtang high-cold steppe zone
		D	Arid zone	HID1	Kunlun high-cold desert zone
HII	Plateau temperate zone	A/B	Humid/sub-humid zone	HIIA/B1	Western Sichuan-eastern Tibet montane coniferous forest zone
		C	Semi-arid zone	HIIC1	Eastern Qinghai-Qilian montane steppe zone
				HIIC2	Southern Tibet montane shrub-steppe zone
		D	Arid zone	HIID1	Qaidam montane desert zone
				HIID2	Northern slopes of Kunlun montane desert zone
				HIID3	Ngari montane desert zone

Table S2. Accuracy evaluation of various snow maps based on in-situ measurements.

In-situ observation	Snow	No-snow	Cloud	Clear Sky Snow accuracy	Clear Sky Overall accuracy	All sky Snow accuracy	All sky Overall accuracy
		MOD10A1					
Snow	138	200	589				
No-snow	109	15000	9456	40.8%	98.0%	14.9%	59.4%
		MYD10A1					
Snow	57	219	643				
No-snow	149	12982	11442	26.0%	97.3%	6.2%	51.1%
		MOYD					
Snow	169	272	478				
No-snow	158	17298	7117	38.3%	97.6%	18.4%	68.5%
		NEARD					
Snow	313	479	127				
No-snow	241	22648	1684	39.5%	97.0%	34.1%	90.1%
		MIMS					
Snow	411	508	-				
No-snow	370	24203	-	-	-	44.7%	96.6%

Supplementary References

1 Paudel, K. P. & Andersen, P. Monitoring snow cover variability in an agropastoral area in the Trans Himalayan region of Nepal using MODIS data with improved cloud removal methodology. *Remote Sensing of Environment* **115**, 1234-1246 (2011).

2 Kostadinov, T. S. & Lookingbill, T. R. Snow cover variability in a forest ecotone of the Oregon Cascades via MODIS Terra products. *Remote Sensing of Environment* **164**, 155-169 (2015).

3 She, J. F., Zhang, Y. F., Li, X. G. & Feng, X. Z. Spatial and Temporal Characteristics of Snow Cover in the Tizinafu Watershed of the Western Kunlun Mountains. *Remote Sensing* **7**, 3426-3445 (2015).

4 Krajci, P., Holko, L. & Parajka, J. Variability of snow line elevation, snow cover area and depletion in the main Slovak basins in winters 2001-2014. *Journal of Hydrology and Hydromechanics* **64**, 12-22 (2016).

5 Lindsay, C., Zhu, J., Miller, A. E., Kirchner, P. & Wilson, T. L. Deriving Snow Cover Metrics for Alaska from MODIS. *Remote Sensing* **7**, 12961-12985 (2015).

6 Gafurov, A. & Bárdossy, A. Cloud removal methodology from MODIS snow cover product. *Hydrology and Earth System Sciences* **13**, 1361-1373 (2009).

7 Parajka, J. & Bloschl, G. Spatio-temporal combination of MODIS images - potential for snow cover mapping. *Water Resources Research* **44,** W03406 (2008).

8 Gao, Y., Xie, H. J., Lu, N., Yao, T. D. & Liang, T. G. Toward advanced daily cloud-free snow cover and snow water equivalent products from Terra-Aqua MODIS and Aqua AMSR-E measurements. *Journal of Hydrology* **385**, 23-35 (2010).

9 Wang, W., Huang, X., Deng, J., Xie, H. & Liang, T. Spatio-Temporal Change of Snow Cover and Its Response to Climate over the Tibetan Plateau Based on an Improved Daily Cloud-Free Snow Cover Product. *Remote Sensing* **7**, 169-194 (2015).

10 Yu, J. Y. *et al.* Developing Daily Cloud-Free Snow Composite Products From MODIS Terra-Aqua and IMS for the Tibetan Plateau. *Ieee Transactions on Geoscience and Remote Sensing* **54**, 2171-2180 (2016).
